# How to Fix Your Forceps before Extracting Teeth

**Published:** 1858-12

**Authors:** H. M. Sheerer


### HOW TO FIX YOUR FORCEPS BEFORE EXTRACTING TEETH.

WELLSVILLE, Alleghany Co., N. Y., March 20, 1858.

MESSRS. EDITORS: In view of the fact, that in our profession, matters of apparently minor significance are quite frequently of considerable importance, I am induced to communicate to you the manner in which I fix my forceps when about to extract bicuspids and molars of either jaw. While performing the operation in question, portions of the lips will frequently work into the joints of the instrument, causing an unpleasant wound of the mouth.

By winding the joints of the forceps with a strip of folded paper or cloth, the occurrence spoken of is effectually prevented.

I wind the joints in this way: Take a strip of cloth or paper, about eight or ten inches long, two inches wide; fold it and wrap around between the handles and beaks, and then around the outside of the joints of the instrument, and tuck the end under.

Your instrument is ready, The winding does not interfere, at all, with the free action of the instrument. Other dentists, perhaps, do this; but there is no harm in speaking about it. Truly, yours, H. M. SHEERER.

---

ELECTRIC ANÆSTHESIA.—Mr. G. Waite, in the London *Lancet* of October 2d, says: "A few years prior to the Great Exhibition of 1851, Mr. Laxton registered for me the right to patent the electro-galvanic current for surgical purposes, its usefulness having struck me when asked by patients to allow them to hold chains, consisting of the metallic combination of various galvanic elements, when undergoing dental operations. Subsequently, at the Great Exhibition, I exhibited battery, with the chain and wires, ready for the dentist's use."

